# Round the Hospitals

**Published:** 1920-07-31

**Authors:** 


### ROUND THE HOSPITALS.

The King held an Investiture in the Quadrangle of Buckingham Palace on July 23, and personally conferred decorations on members of the Nursing Services as follows: *The Most Excellent Order of the British Empire, Members, Civil Division*: Miss Edith Spencer, who also received the Royal Red Cross, Second Class, and others. *Royal Red Cross, Bar*: Miss Ethel Denne, Q.A.I.M.N.S.; Miss Mary Fisher, V.A.D. *First Class*: Misses Eva Mason, Alice Rowe, Christine Sandbach, Q.A.I.M.N.S.; Misses Ethel Beloe, Elsie Evans, Mary Gregory, Maud Hopton, Winifred Hughes, Emily Middlemist, Hannah Henderson-Smith, Edith Stanton, Mrs. Netta Dunlop, Q.A.I.M.N.S.R.; Mrs. Phœbe Balmforth, Misses Martha Reid-Morison, Katharine Todd, T.F.N.S.;

**ROUND THE HOSPITALS**—(*continued*).

Misses Lucy Ellis, Lucy Garnet, Rachel Paterson, C.N.S.; Mrs. Frances Stephens, B.R.C.S.; Misses Maud Beverley and Jessie Robertson, C.H.R.; Miss Mary Tyndall, C. and W. Hosps. *Second Class*: Miss Bertha Martin, Q.A.R.N.N.S.; Misses Dora Mason, Margaret Riddell, Jean Todd, Q.A.I.M.N.S.; Misses Margaret Houston, Jessie Macqueen, Nina Maling, Edith Marshall, Christina McDonald McLennan, Alice Rennison, Edith Smith, Q.A.I.M.N.S.R.; Misses Nellie Hayes, Hilda Purse, Eliza Robertson, Violette Rutter, Maud Stainton, T.F.N.S.; Misses Annie Gardner, Agnes Greenshields, Mary Millar, Florence Whitley, C.N.S.; Misses Ethel Florence, Hannah Gobbett, Emma Jay, Bessie Smith, Ellen Surman, Mrs. Amy Skinner, B.R.C.S.; Miss Elizabeth C.H.R.; Miss Olive Plummer, C. and W. H.; Miss Ethel Pratt, Uganda N.S.; Misses G. Bell, V. Collett, K. Firth, M. Harding, G. Morris, W. Spencer, M. Stout, M. Tracy, C. Walker, S. Watson, E. Welber, Mrs. E. Hardiman, Mrs. M. Peat, V.A.D. Queen Alexander received at Marlborough House all the above ladies after the Investiture.

In the absence of the King at the funeral of the Empress Eugénie on July 20, the Duke of York held an Investiture on behalf of His Majesty in the Quadrangle of the Palace, when the following members of the nursing services received the Order of the Royal Red Cross:—*Bar*: Miss Maud MacDonnell, B.R.C.S. *First Class*: Miss Flora Craig, Q.A.I.M.N.S.; Mrs. Alice de Winton, Miss Christina Soutar, and Miss Scott Newton, Q.A.I.M.N.S.R.; Mrs. Constance Pigg, V.A.D. *Second Class*: Miss Daisy Martin, Q.A.I.M.N.S.; Miss Patricia Meany, Miss Susan Bradshaw, Miss Agnes Campaigne, Miss Ellen Disney, Miss Dora Fraser, Miss Christina Henderson, Miss Kathleen McLean, Miss Alice Moxon, Miss Elinor Roberts, and Miss Dinah Steele, Q.A.I.M.N.S.R.; Miss Edith Denison, Miss Amelia Dobson, and Miss Olive Greenwell, T.F.N.S.; Miss Mary Whyte, C.N.S.; Myfanwy Lady Hoskins, Miss Agnes Ormiston, and Miss Dorothy Philpott, B.R.C.S.; Miss Bessie Reid, C.H.R.; Miss Ida Fyson, Miss Joan Husey, Miss Maud Kirk, Miss Jane Leresche, Mrs. Nora McPellan, Miss Millicent Norton, and Miss Dorothy Ridley, V.A.D.

The Army Council announces that it has prepared an inscribed scroll, to be hung in all buildings, if so desired, which were used as hospitals for the sick and wounded during the war. The total number of buildings adapted for use as hospitals was 3,244, of which the largest numbers, 687, 660, and 637 respectively, were situated in the Eastern, Southern, and Western Commands. The scroll record that the building was established and maintained as a hospital for British sick and wounded during the Great War of 1914-1919. It continues: "The Army Council, in the name of the nation, thanks those who have rendered to it this valuable and patriotic assistance in the hour of its emergency, and they desire also to express their deep appreciation of the whole-hearted attention which the staff of this hospital gave to the patients who were under their care. The war has once again called upon the devotion and self-sacrifice of British men and women, and the nation will remember with pride and gratitude their willing and inestimable service." More than 500 scrolls have already been issued.

The Presidential address at the Conference on health visitors at the Birmingham Congress of the Royal Sanitary Institute last week was given by Miss H. S. Cooper-Hodgson, Superintendent Health Visitor, Durham County Council. She gave a highly interesting account of the intricate and important work carried out in the county of Durham in the course of the year by the fifty health visitors, and the cordial manner in which their ministrations were accepted. She deprecated the tendency on the part of doctors and others to stir up discord between midwives, district nurses, and health visitors who "are *not* engaged in quarrelling with each other." Miss Cooper-Hodgson had much to say about the best training for health visitors, and we regret to find that she does not advise the would-be health visitor to spend three years in a general hospital. She believes that fully-trained nurses have acquired a bias which she knows to be detrimental, that their predominant feeling is for the treatment of disease and not for prevention. We believe her experience to have been unfortunate in this respect, and her conclusions to be erroneous. But we believe also that many training-schools have something to answer for in fixing the minds of the probationers too exclusively on disease, and that this tendency is best counteracted by the admission of women to training whose future work is to be preventive rather than remedial or curative.

A noteworthy feature of the King's Fund distribution of the Red Cross surplus funds, reported on another page of this issue, is the ear-marking of portions of many of the grants for the purpose of extensions or improvements to the Nurses' Home. This is a natural outcome of the movement for shorter hours of working and, consequently, larger staffs, and of the Fund's recognition of the importance of housing nurses properly if they are to do their work efficiently. The Distribution Committee, in its report, refers to the extent to which the success of medical and surgical treatment depends on nursing. No fewer than twenty-three hospitals receive grants towards improvements in the accommodation provided for nurses, the largest sum being £24,000 to St. Bartholomew's Hospital.

An important decision was reached at the recent meeting of the Council of Queen Victoria's Jubilee Institute at which Sir W. Cameron Gull presided. This was no less than an immediate increase of salary and allowances to all the Queen's nurses,

**ROUND THE HOSPITALS**—*(continued).*

and it followed on a recommendation from a conference of representatives of the affiliated associations in England and Wales. The new minimum rate agreed upon is £63, rising by annual increment to £75, with further increases according to the qualifications of the nurses and the work undertaken. Superintendents and their assistants, as well as senior nurses, will be paid at higher rates. The minimum allowance for board and personal laundry has been raised to 25s. a week. Uniform allowance is to be £10 a year; and, in addition, nurses are to be provided with two furnished rooms, and with fire, light, and attendance. Thus the above salary will be clear money. The decision was received with great satisfaction by the Council. The annual report indicated that the work cannot in effect be carried on unless associations are prepared to furnish their nurses with adequate pay. Last year there were 324 resignations, while the number of nurses newly-placed on the Roll amounted to only 121. Even including the seventy former Queen's nurses who rejoined, there was a diminution of 131 nurses on the Roll in the one year's working. It is useless to go on training more women to become district nurses if they find on trial that they cannot get a living by this work. The new regulations should give a great impetus to the organisation.

No fewer than twenty matronships have been gained by Guy's nurses during the past year. The list of appointments gained includes posts in England, Scotland, India, Persia, Egypt, Canada, Rhodesia, and South and East Africa. The Guy's Hospital report records the fact that two of the governors have provided £3,200 for the enlargement and improvement of the Nurses' Cottage at Honor Oak as a memorial and recognition of the services rendered to their country by Guy's nurses in the war. The summary of honours gained by the Guy's nurses makes good reading: Royal Red Cross: Bar, 1; First Class, 29, Second Class, 88. French, Belgian, Greek, Serbian, Italian, and Russian medals, 15; Military Medals, 5; Albert Medal, 1; Special Services Cross, 1. Mentioned in despatches, 50; mentioned for valuable services in home hospitals, 38. In addition, Miss Gladys Laura White, R.R.C., formerly surgery sister, was awarded the Florence Nightingale Medal by the International Red Cross Committee at Geneva for services 1915 to 1918, especially at No. 9 B.R.C.C., at St. Omer.

In South Wales and elsewhere it is proving very difficult to attract the right kind of women to take training as village midwives, and the County Medical Officer pleaded at the meeting of the Carmarthenshire Public Health Committee for trainees from the ranks of married people and widows who had a strong local attachment. He finds that the nurses trained by the South Wales Nursing Association take up school and health visiting in preference to the more arduous midwives' work, and mothers are left to the ministrations of untrained women. If women with homes of their own are to be attracted to take up midwifery the training-schools for village nurses must make concessions and arrange such conditions of living whilst in training as will not repel them from the work. Also a much more intelligent form of appeal must be set in motion among the villages.

After a lengthy inquiry the Committee of the Board of Guardians appointed to enquire into the administration of the Merthyr Tydfil Infirmary have unanimously reported as follows:—"That in the opinion of the Committee there is a certain slackness and lack of discipline in the Infirmary, very largely due to the want of tact on the part of the superintendent and certain officers of the institution. That there are grounds for complaint as to the manner in which the meals are served. That the superintendent nurse be called upon to pay more attention to the mess-room, and that the superintendent or one of the sisters must preside over all meals. That the statement that Sister Elvina Davies spoke slightingly of the probationers is proved, and in view of her statements and conduct before the Inquiry Committee she be called upon to resign." It seems very doubtful whether these suggestions go to the root of the matter. The nurses have evidently been very uncomfortable, and the Guardians should appoint a small nursing committee charged with the duty of carrying out improvements in consultation with the superintendent nurse.

The Council of the Nightingale Fund has elected Miss Olive Baggalay, Miss Margery Cave, and Miss Katherine Thornton Down to Nightingale scholarships at the household and social science department of King's College for Women for 1920-21. The demand for sister tutors continues to be greater than the supply. We note with satisfaction that under the new scale of salaries sanctioned by the Metropolitan Asylums Board in June the remuneration of the sister-tutor at Queen Mary's Hospital is fixed at £130, or, if specially qualified after taking a course of instruction, £156.

The Committee of the South London Hospital for Women and Children, Clapham Common, has appointed Miss K. E. Luard, R.R.C., to the vacant post of matron. Miss Luard, who distinguished herself in the war, was trained at King's College Hospital, and later became out-patient sister at the Evelina Hospital for Sick Children. From there she went to Charing Cross Hospital as night superintendent, resigning that post to become matron of the Bucks and Berks Joint Sanatorium, Peppard Common, Oxon. During the war Miss Luard served in France in Queen Alexandra's Imperial Military Nursing Service Reserve, was twice mentioned in despatches, and received a Bar to her Royal Red Cross.

Miss K. G. Lloyd, R.R.C., assistant matron at the General Hospital, Birmingham (whose training and career we give on another page), has been

**ROUND THE HOSPITALS**—(*continued*).

appointed matron of the Royal Lancaster Infirmary, out of over forty applicants. Miss Lloyd succeeds Mrs. Crewe, who has been matron at the Royal Lancaster Infirmary for thirty-nine years, during which she has seen a new and up-to-date infirmary built, the work trebled, and the whole system of hospital treatment practically revolutionised. The institution now contains eighty beds (ten in the children's ward), seventy-five being occupied on an average. There is a "light" room, with $x$-ray and electrical treatment, and an orthopædic clinic has just been established. The present infirmary was opened in 1896 by the present King and Queen (then Duke and Duchess of York), who conveyed the command of the late Queen Victoria that the prefix "Royal" was to be used. Since they came to the Throne the King and Queen have graciously become patrons of the institution.

---

The 3rd London General Hospital on Wandsworth Common, of which Miss Edith Holden has been matron since it opened till the last day, closed down on July 21, and a cheerful gathering of all who still remained in residence, including both patients and staff, marked the occasion. It will be remembered that this great establishment was formed in and around the Royal Patriotic School. In six years it has treated over 62,000 patients and received men for treatment for every one of the Allies, including, of course, a very large number from the Dominions. Its successful administration and the admirable quality of the nursing are demonstrated by the fact that the deaths were less than 1 per cent.

---

A delightful concert was given, in aid of St. Bartholomew's Hospital, under the presidency of Viscount Sandhurst, at 10 Downing Street, on July 21, by permission of Mr. and Mrs. Lloyd George, the latter of whom, with Miss Megan Lloyd George, was present, while the Prime Minister appeared for a few moments in the doorway to hear one of Madame Elsa Stralia's songs. A very distinguished gathering came together to hear the music and to assist at the mock auction which took place in the course of the afternoon. Mr. Alfred T. Davies, M.P., was the auctioneer, helped out by Mr. Tom Burke, the well-known singer, and all manner of gifts presented for the Bart's Fund were disposed of to great advantage, including a cow and an ambulance flag from Jerusalem, which fetched £205.

---

The Welsh Priory of the Order of St. John has set up a scheme in Cardiff which deserves widespread imitation. It is for the opening of depôts in various districts for the sale at nominal cost to the sick poor and disabled soldiers of medical comforts, such as hot-water bottles, rubber beds, spinal carriages, etc., with wheel-chairs on hire. On the opening of the first depôt a large crowd flocked to take advantage of the goods, which were under the charge of the nurses. The closing down of the Welsh war hospitals enabled the secretary, Mr. Herbert Lewis, to secure medical equipment on very easy terms. Patients who desire to benefit under the scheme must provide themselves with coupons from medical men. Two of the nursing members of the Order of St. John give attendance daily, and the work is under the superintendence of the Principal Matron to the Priory, Mrs. E. M. Owen.

---

The visit of the King and Queen to Newington House, the Scottish Blinded Soldiers' and Sailors' Institution, was rendered memorable by the demonstration afforded of the optophone, the magical instrument through which ordinary print can be translated into sound for the use of the blind. The King was deeply impressed by this "simply marvellous" invention, the work of Dr. E. Fournier d'Albe, modified and developed by Messrs. Barr and Stroud, of Glasgow. It is a small machine, producing in a telephone receiver a series of musical notes. Each letter of print or typescript gives a different sound as it passes over the machine, and by practice the listener apprehends the sound message delivered to the ear as rapidly as the sighted person apprehends the message to the eye. The demonstration given by Emeritus Professor Archibald Barr was followed with deep interest by the Royal party.

---

Last week Mrs. Copeland, a widow and former sanitary inspector at Ealing, was awarded by the jury £25 in a slander action against Dr. Thomas Orr, medical officer to the Ealing Borough Council. The case came up before Mr. Justice Roche, who said there was just such evidence that he could not properly reverse the jury's decision, and he therefore entered judgment for plaintiff for £25. At the same time he added, "I don't see how a doctor under a public authority can carry on his work if actions of this sort are brought and succeed on such evidence." Mrs. Copeland brought the action on the ground that Dr. Orr had slandered her by telling the borough council that her methods as sanitary inspector and health visitor were bad and slovenly, and that when she was tackled about it she burst into tears and took herself off for three days. It appears that she lost her post in consequence of this report. Stay of execution pending appeal was granted. The result will be awaited with much interest; it is a case of the utmost importance to all public authorities employing health visitors.

---

The lay press in New South Wales is valiantly pleading the cause of the nurses and exposing the hard lot of their "modest and noble sisters." The fact that nurses in Victoria have been placed by law under the eight-hour system has thrown into strong relief the very different conditions under which the average nursing sister works in other parts of Australia, and we wish the reformers all success. The contrast between the official amount of leave and what is actually obtained is startling, and this is an abuse which it is very difficult to overcome except by legislation.

